# Consumption—A Suggestion

**Published:** 1854-12

**Authors:** 


### CONSUMPTION—A SUGGESTION.

In November and December of each year multitudes bear away to the sunny south and to the isles of the sea, leaving behind them dear homes which, in many instances, they shall never see again, sundering associations and ties and hearts and loves, to be reunited no more. Some, and not a few, find in a few days after they reach those sunny climes, that the flowers do indeed bloom as in the springtime, the birds sing as gladsomely, and the clear blue sky and the bright warm sunshine bring gladness and health to all—but themselves; that the chill blood in their veins is not warmed, the hectic in their cheek is not dissolved into the red hue of health, while the song of the bird and the fresh tint of the flower carry them back to their childhood's home, now far, far away, and the one ambition now is, to go and die at home. They find that the mild, warm weather of the south debilitates them just as much as their own summers—why did they not think of that before? Could a warm day in latitude twenty impart any influences more than an equally warm day in latitude forty would do? This question is discussed at length in the eighth edition of Bronchitis and Kindred Diseases, (one of Redfield's publications, see last page of cover.)

If a man is sick and must leave home, the general suggestion would be to select the most healthful locality. Vermont is the healthiest State in the Union, and the records of mortality for the last thirty years in the cheery city of Portland, Maine, justify the assertion that it is the healthiest city on this continent. The proof is in the fact among others, that it has never been visited by the cholera or suffered from any alarming epidemic. In spite of its proximity to the ocean, it must be a delightful resort for consumptives at least during the hot summer months, which make such large drafts upon the strength of all invalids.

